# Transcriptional Response of Zebrafish Embryos Exposed to Neurotoxic Compounds Reveals a Muscle Activity Dependent *hspb11* Expression

**DOI:** 10.1371/journal.pone.0029063

**Published:** 2011-12-19

**Authors:** Nils Klüver, Lixin Yang, Wibke Busch, Katja Scheffler, Patrick Renner, Uwe Strähle, Stefan Scholz

**Affiliations:** 1 Department of Bioanalytical Ecotoxicology, UFZ - Helmholtz Centre for Environmental Research, Leipzig, Germany; 2 Institute of Toxicology and Genetics, Karlsruhe Institute of Technology, Eggenstein-Leopoldshafen, Germany; University of Sheffield, United Kingdom

## Abstract

Acetylcholinesterase (AChE) inhibitors are widely used as pesticides and drugs. Their primary effect is the overstimulation of cholinergic receptors which results in an improper muscular function. During vertebrate embryonic development nerve activity and intracellular downstream events are critical for the regulation of muscle fiber formation. Whether AChE inhibitors and related neurotoxic compounds also provoke specific changes in gene transcription patterns during vertebrate development that allow them to establish a mechanistic link useful for identification of developmental toxicity pathways has, however, yet not been investigated. Therefore we examined the transcriptomic response of a known AChE inhibitor, the organophosphate azinphos-methyl (APM), in zebrafish embryos and compared the response with two non-AChE inhibiting unspecific control compounds, 1,4-dimethoxybenzene (DMB) and 2,4-dinitrophenol (DNP). A highly specific cluster of APM induced gene transcripts was identified and a subset of strongly regulated genes was analyzed in more detail. The small heat shock protein *hspb11* was found to be the most sensitive induced gene in response to AChE inhibitors. Comparison of expression in wildtype, *ache* and *sop^fixe^* mutant embryos revealed that *hspb11* expression was dependent on the nicotinic acetylcholine receptor (nAChR) activity. Furthermore, modulators of intracellular calcium levels within the whole embryo led to a transcriptional up-regulation of *hspb11* which suggests that elevated intracellular calcium levels may regulate the expression of this gene. During early zebrafish development, *hspb11* was specifically expressed in muscle pioneer cells and Hspb11 morpholino-knockdown resulted in effects on slow muscle myosin organization. Our findings imply that a comparative toxicogenomic approach and functional analysis can lead to the identification of molecular mechanisms and specific marker genes for potential neurotoxic compounds.

## Introduction

Understanding of chemical-induced biological responses and their underlying pathways is indispensable to evaluate the impact of chemicals on organisms. Toxicogenomic analysis is an effective approach to better understand these pathways and their related adverse effects [1]–[3]. A major step forward is the identification of chemical-specific gene expression signatures which could be used to assign mechanisms of action (MoA) to non-characterized or unknown compounds [4]. Changes in gene expression by exposure to chemicals can be directly inferred from binding of a compound to a transcription factor. Prominent examples are the aryl hydrocarbon or estrogen receptors. However, many compounds, e.g. neurotoxic chemicals, may not interfere directly with a transcription factor but affect gene expression patterns indirectly through a complex signaling cascade.

In the present study, we examined the toxicogenomic response to azinphos-methyl (APM), an AChE-inhibitor, using zebrafish embryos as a model. AChE catalyzes the hydrolysis of the neurotransmitter acetylcholine (ACh) and is important for the cholinergic neurotransmission. Furthermore, non-cholinergic functions of AChE have been described in neuronal development [5], [6]. AChE is the target of many toxins like insecticides, chemical weapons, and snake venoms [7]. Prolonged AChE inhibition or complete lack of AChE results in an accumulation of ACh and overstimulation of the muscle, leading to spasms and myopathy-like phenotypes [5], [8], [9]. The zebrafish embryo has been selected as model for the following reasons: (i) Toxicogenomic approaches in the zebrafish embryo have recently shown that chemicals can provoke specific and sensitive gene expression changes [10]–[12]. (ii) It has been described that AChE is required for neuronal and muscular development in zebrafish embryos and no other ACh-hydrolyzing enzymes are present in this organism [5], [13], [14]. (iii) Appropriate mutant strains that either lack AChE (*ache*) or nAChR (*sop^fixe^*) activity are available to support functional analyses.

First, we compared changes in gene expression patterns provoked by APM with those of two non-AChE inhibiting compounds, 1,4-dimethoxybenzene (DMB), a narcotic (baseline toxic) compound and 2,4-dinitrophenol (DNP), an oxidative decoupling agent. The test compounds were selected to allow the identification of genes specifically responding to compounds inhibiting acetylcholine esterase. Neurotoxicity is a common MoA among compounds exhibiting an excess toxicity (i.e. ratio of predicted baseline toxicity versus measured toxicity). The organophosphate insecticide and known acetylcholine esterase inhibitor APM [15] exhibits a high acute excess toxicity, i.e. the measured LC_50_ is 1340fold below the predicted acute toxicity [16]. This clearly supports the specific neurotoxic mode of action. The other compounds were selected as two different non-neurotoxic reference compounds in order to allow the identification of MoA specific gene expression patterns. DNP, an industrial chemical, is a protonionophor or uncoupling agent that disrupts generation of ATP from proton-gradients in the mitochondria [17]. DNP has a moderate excess toxicity of 23. In contrast to APM and DNP the acute fish toxicity of DMB is in the same order of magnitude as the predicted baseline-toxicity. Subsequently, detailed transcriptional analyses of selected genes were applied to link gene expression changes to the MoA. We investigate the expression of the small heat shock protein *hspb11* in wildtype, *ache* and *sop^fixe^* mutant embryos in more detail and study its role during development. For the first time, we demonstrate that developmental *hspb11* expression is muscle-activity dependent and is indirectly regulated by intracellular calcium levels. Furthermore, Hspb11 is required for slow muscle myofibril organization in the embryo. These results show that a comparative toxicogenomic approach and functional analysis can lead to the identification of molecular mechanisms and specific marker genes for potential neurotoxic compounds.

## Results

### Toxicogenomic responses to APM exposure are highly specific

**Figure 1:**
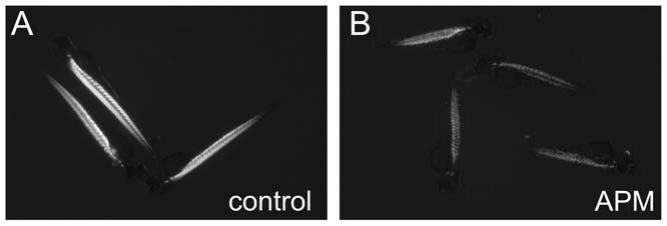
Repression of AChE activity with APM resulted in a reduced birefringence. (A) Wild-type zebrafish embryo axial musculature is highly birefringent at 72 hpf. (B) APM [6 µM] exposure from 12–72 hpf resulted in defects in the axial musculature, which are shown by a reduced birefringence at 72 hpf.

For transcriptional profiling it was aimed that similar – with respect to toxicity – effect concentrations were used that did not induce severe morphological changes. Based on concentration response curves of zebrafish embryos exposed from 2-50 hours post fertilization (hpf) we selected the modeled LC_10_ (6 µM for APM, 509 µM for DMB and 14 µM for DNP) as exposure concentrations for the microarray study (Figure S1). Exposures to this effect level did not induce gross morphological changes. However, APM inhibited zebrafish AChE enzymatic activity half-maximally at 0.15 µM and caused an almost complete block of AChE activity at 6 µM (Figure S2). It is known that in *ache* mutants the lack of AChE activity can cause a progressive myopathy in zebrafish embryos [5], [8]. To test whether APM caused similar defects we performed a birefringence analysis of embryos exposed from 12–72 hpf to 6 µM APM (according to [8]). APM treated embryos were immobile and displayed a reduced birefringence compared to controls (Figure 1). This suggests that a prolonged exposure with 6 µM APM caused changes in muscular structures.

For the microarray analysis we exposed zebrafish embryos for 24 h (26–50 hpf) and 48 h (2–50 hpf). The different exposure durations were chosen in order to identify potential differences with respect to primary or secondary responses. By hierarchical cluster analysis we identified an APM specific cluster of significantly induced genes after the 24 h as well as the 48 h exposure (Figure S3). The observed differences, however, were more prominent after 24 h of exposure. In order to identify pathways, associated with the transcriptional changes, a gene set enrichment analysis (GSEA) was performed. Some of the identified gene sets in the 24 h and 48 h treatments referred to factors/genes involved in neuronal and muscle function/differentiation (axon guidance, neurogenesis, voltage gated channel activity, muscle structure and contraction, muscle tissue maintenance and differentiation, supplement Table S1). More gene sets indicating effects specific for neurons and muscles were identified for the 48 h treatment. This may indicate that GSEA revealed (subtle) changes in neuron or muscle structure and differentiation. However, GSEA did not indicate interference with a specific signaling pathway.

### Transcriptional changes of the zebrafish embryo are AChE-inhibitor concentration-dependent

**Figure 2:**
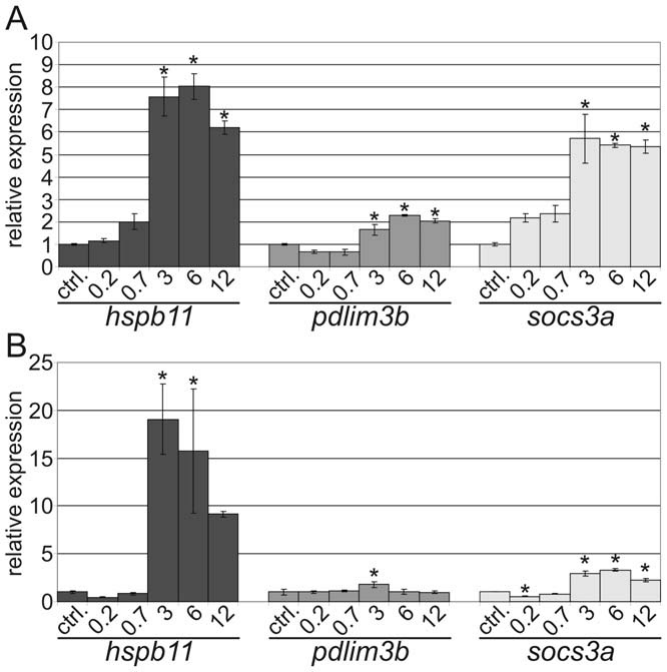
APM concentration dependent induction of *hspb11*, *pdlim3b* and *socs3a* in zebrafish embryos. APM exposures for (A) 24 h (26–50 hpf) and (B) 2 h (48–50 hpf). Concentrations are given in µM. Bars represent the relative gene expression as fold change of the respective untreated control as mean ± standard deviation of three independent replicate exposures. Control  =  ctrl. * P<0.05.

In order to unravel the MoA leading to the expression changes in the APM-specific gene cluster, we selected *hspb11* (heat shock protein, alpha-crystallin-related, b11), *pdlim3b* (PDZ and LIM domain 3b), and *socs3a* (suppressor of cytokine signaling 3a) for concentration-dependent quantitative expression analysis. The selection of these genes was based on an initial RT-PCR verification experiments (not shown) and the genes covered different potential gene functions, e.g. stress response (*hspb11*), immuno response (*socs3a*) and cytoskelatal assembly (*pdlim3b*). The expression of these genes was gradually increased with elevated APM concentrations. By means of qPCR an about two-fold (*pdlim3b*), 8-fold (*hspb11*) and 6-fold (*socs3a*) maximum induction after 24 h of exposure was observed (Figure 2A). Lowest observed effect concentrations for gene expression changes compared to the control were 0.7 µM and 3 µM of APM. Changes of *hspb11* and *socs3a* gene expression were already detected in 2 h (48 to 50 hpf) APM exposures whereas the induction of *hspb11* (approx. 20-fold) exceeded the levels of expression after 24 h of exposure (Figure 2A and B). The expression of *hspb11* was also found to be induced by other AChE inhibitors such as propoxur, disulfoton, and galantamine (Figure S4). In contrast, the expression of *socs3a* was not affected by the disulfoton treatment but also elevated after a treatment with the non-AChE-inhibiting compound 4-nitrophenol (Figure S4D). *Pdlim3b* was induced by all tested AChE inhibitors and not by 4-nitrophenol. This suggests that *hspb11* and *pdlim3b* up-regulation is a specific response to AChE inhibitors. Due to the strong and sensitive changes in *hspb11* expression subsequent functional analyses were focused on *hspb11*.

### APM Mediated *hspb11* Up-Regulation Depends on nAChR Activity

**Figure 3:**
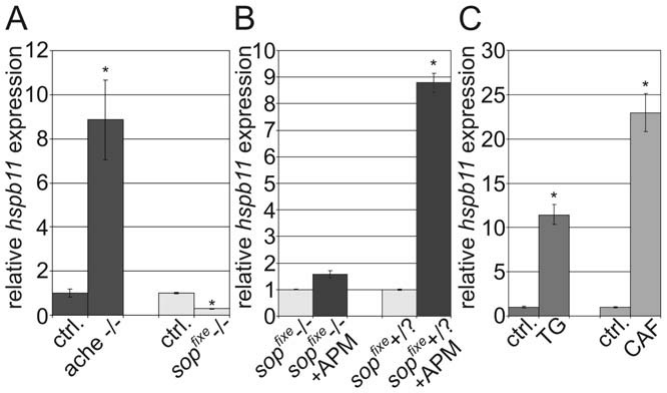
APM mediated *hspb11* induction depends on nAChR activity and increased intracellular calcium levels. (A) *hspb11* expression analysis in *ache* and *sop^fixe^−/−* zebrafish mutant embryos at 48 hpf. (B) *hspb11* expression in *sop^fixe^* null mutants and siblings (+/?  =  heterozygous and wildtype) after APM (6 µM) exposure (26–50 hpf). (C) 1 µM Thapsigargin (TG) and 2 mM caffeine (CAF) induce *hspb11* expression in zebrafish embryos (exposure period 48–50 hpf). Bars represent the relative gene expression as fold change of the respective untreated control as mean ± standard deviation of three independent replicate exposures Control  =  ctrl. * P<0.05.

APM-induced *hspb11* expression could be mediated by the overstimulation of the nAChR. In order to elucidate the role of nAChR we analyzed *hspb11* expression in the zebrafish *ache* and *sop^fixe^* mutant lines. Due to a point mutation in the *ache* gene, homozygous *ache* mutant embryos completely lack AChE activity [5]. The *sop^fixe^* mutant harbors a point mutation in the δ-subunit of the muscular nAChR, encoded by the gene *chrnd*, and, hence, lacks a functional nAChR. The δ-subunit is expressed in all striated muscles in embryonic and early larval stages [18]. Homozygous (−/−) forms of both mutant embryos show severe impairment of motility but are viable up to 5 days post fertilization (dpf). Interestingly, the expression of *hspb11* was strongly increased in 48 hpf *ache* −/− and repressed in *sop^fixe^* −/− mutant embryos (Figure 3A). Furthermore, exposure of *sop^fixe^* −/− with APM (24–48 hpf) did not result in further induction of *hspb11* expression, whereas *sop^fixe^* siblings exposed to APM showed a 9-fold up-regulation (Figure 3B). These data indicate that *hspb11* induction by APM is dependent on a functional nAChR.

### Modulators of Cytosolic Calcium Levels Induce *hspb11* Expression

ACh triggers muscular contraction by depolarizing muscle cells and subsequent transient release of calcium from the sarcoplasmatic reticulum. Calcium is not only the principal signaling molecule for muscle contraction, it is also required for normal development of muscles. It is hypothesized that excess activation of AChR by unhydrolyzed ACh results in a large influx of calcium ions in muscle cells. Indirect evidence for the role of calcium ions is provided by studies that demonstrate a rescue of myopathy (provoked by exposure to AChE inhibitors) by calcium channel blockers [19]–[21]. Hence, we concluded that *hspb11* induction by AChE inhibition might be a result of increased cytosolic calcium levels and increased muscular contractions mediated by overstimulation of the nAChR. Hence, non-AChE inhibiting compounds that lead to an increase in cytosolic calcium should increase *hspb11* mRNA abundance. Therefore, we exposed zebrafish with thapsigargin and caffeine. Thapsigargin is a specific blocker of the sarco/endoplasmic reticulum calcium-ATPase (SERCA) [22]. Caffeine represents an activator of ryanodine receptors (RyRs), a major mediator of calcium-induced calcium release in adaxial muscles [23]. Exposure of wildtype embryos with either thapsigargin [1 µM] for 2 h (48–50 hpf) or caffeine [2 mM] for 24 h (26–50 hpf) resulted in an increased *hspb11* gene expression. Thapsigargin induced *hspb11* mRNA expression 11-fold and caffeine 23-fold (Figure 3C).

### *Hspb11* Is Expressed in Muscle Pioneers and Shows a Muscle-activity Dependent Expression

**Figure 4:**
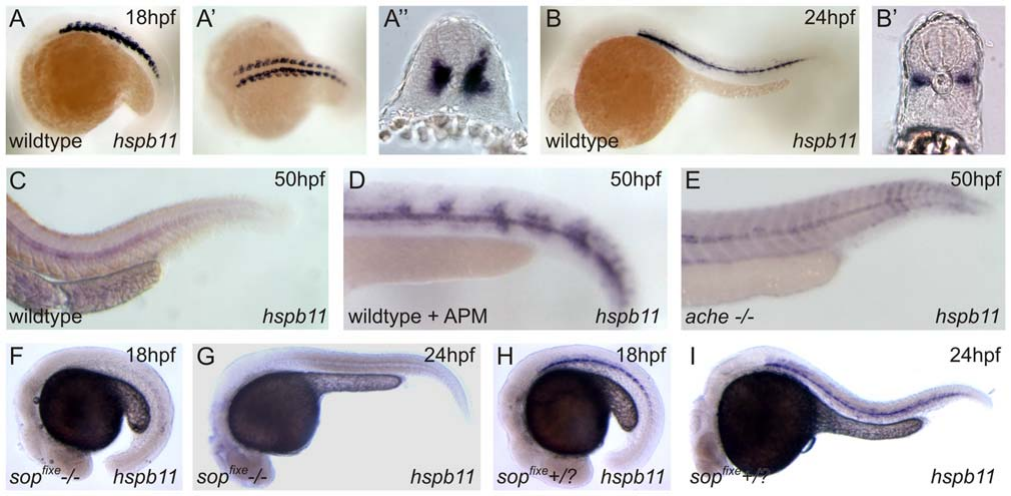
Developmental *hspb11* expression pattern in wildtype, APM exposed, *ache* and *sop^fixe^* mutant embryos. Expression pattern of *hspb11* at 18 hpf were detected in developing adaxial musculature (A–A″). During later developmental stages, at 24 hpf, *hspb11* mRNA transcripts were restricted to muscle pioneers (B and B′) and became only expressed in the notochord at 50 hpf (C). (A, B) lateral view, (A′) dorsal view and (A″ and B′) cross sections. Increased expression pattern in axial musculature was observed in APM exposed (D) and homozygous (−/−) *ache* mutant embryos (E). *Hspb11* expression in homozygous *sop^fixe^−/−* embryos (F and G) and heterozygous/wildtype (+/?) siblings (*H* and *I*).

**Figure 5:**
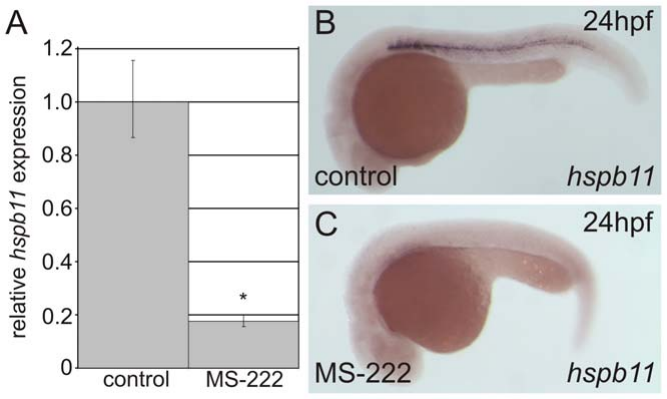
Blocking the skeletal muscle contraction with MS-222 inhibits developmental *hspb11* expression. (A) qPCR *hspb11* expression analysis. (B) Wildtype *hspb11* expression pattern at 24 hpf. (C) MS222 treatment results in the loss of *hspb11* expression pattern at 24 hpf.

In order to determine the function of *hspb11*, we first analyzed the spatiotemporal *hspb11* expression. At 18 hpf *hspb11* is expressed in the adaxial cells (i.e. somitic cells next to the notochord, Figure 4A–A″). At 24 hpf *hspb11* mRNA expression is restricted to muscle pioneers (Figure 4B and B′). To elucidate whether the *hspb11* transcripts are exclusively expressed at these early stages in muscle pioneers, we screened the *hspb11* expression pattern in *smu −/−* mutant embryos. *smu* mutants are defective in smoothened - a mediator of hedgehog signals - and lack muscle pioneers [24]. In *smu −/−* embryos *hspb11* expression was lost (Figure S5A and B) indicating that *hspb11* is specifically expressed in muscle pioneers. During later development *hspb11* expression was restricted to the notochord (Figure 4C). Interestingly, this *hspb11* expression pattern changed in APM exposed embryos and in *ache−/−* mutant embryos where transcripts were also detected in the myotomes of the trunk in addition to the notochord (Figure 4D and E). To test whether the developmental *hspb11* expression is dependent on nAChR activity we investigated the *hspb11* expression pattern of *sop^fixe^* mutants. Of a single cross of heterozygote parents we used 30 embryos at 18 hpf for WISH. In 8 embryos, we did not detect any *hspb11* expression pattern in muscle pioneers whereas 22 embryos showed a wildtype *hspb11* expression pattern, as expected for a recessive trait inherited in a mendelian fashion (Figure 4F and H). Further, at 24 hpf the *sop^fixe^ −/−* embryos lack *hspb11* expression pattern whereas *sop^fixe^+/?* embryos have a wildtype *hspb11* expression pattern (Figure 4G and I). To exclude that the lack of *hspb11* expression pattern is caused by a loss of muscle pioneers in *sop^fixe^−/−* embryos, we analyzed the *eng2a* expression in *sop^fixe^−/−* embryos. It is known that muscle pioneers highly express *engrailed1* and *engrailed2* genes [25]. In all embryos of a cross of heterozygous parents, we detected muscle pioneer specific *eng2a* expression at 18 hpf (Figure S5C). These altered *hspb11* expression patterns led us to ask if the *hspb11* expression is muscle-activity dependent and can be repressed by blocking the muscle contraction with the anesthetic MS-222, a voltage-gated Na+ channel blocker, in order to prevent action potentials. Treatment with MS-222 [0.5 mM] for a 6 h (18 hpf to 24 hpf) resulted in a significant *hspb11* repression (Figure 5A) and the wildtype *hspb11* expression pattern was lost (Figure 5B and C). This suggests that the developmental *hspb11* gene expression is muscle activity dependent.

### Hspb11 is Involved in Slow Muscle Myosin Organization and/or Maintenance

To examine the developmental function of Hspb11, we performed a morpholino-mediated knockdown of Hspb11. Therefore we used two translation-blocking morpholinos, MO(ATG)-*hspb11* and MO(5′UTR)-*hspb11*, and a mismatch control morpholino (mmMO-*hspb11*) that harbors five mismatches compared to the MO(*ATG*)-*hspb11* were used. To avoid interference with nonspecifically induced apoptosis a morpholino directed against *p53* was co-injected [26]. To prove that the injected *hspb11* morpholinos are specific to *hspb11* mRNA we performed additional control experiments. First, we injected a *gfp* mRNAs that contains either the morpholino binding site for MO(ATG)-*hspb11* or MO(5′UTR)-*hspb11* upstream of the GFP coding sequence and we detected a bright GFP fluorescence (Figure S6). Coinjection of *gfp* mRNA with either MO(ATG)-*hspb11* or MO(5′UTR)-*hspb11* strongly reduced the GFP fluorescence (Figure S6). Further, coinjection with the designed mmMO-*hspb11* and *gfp* mRNA did not reduce the GFP fluorescence (Figure S6).

**Figure 6:**
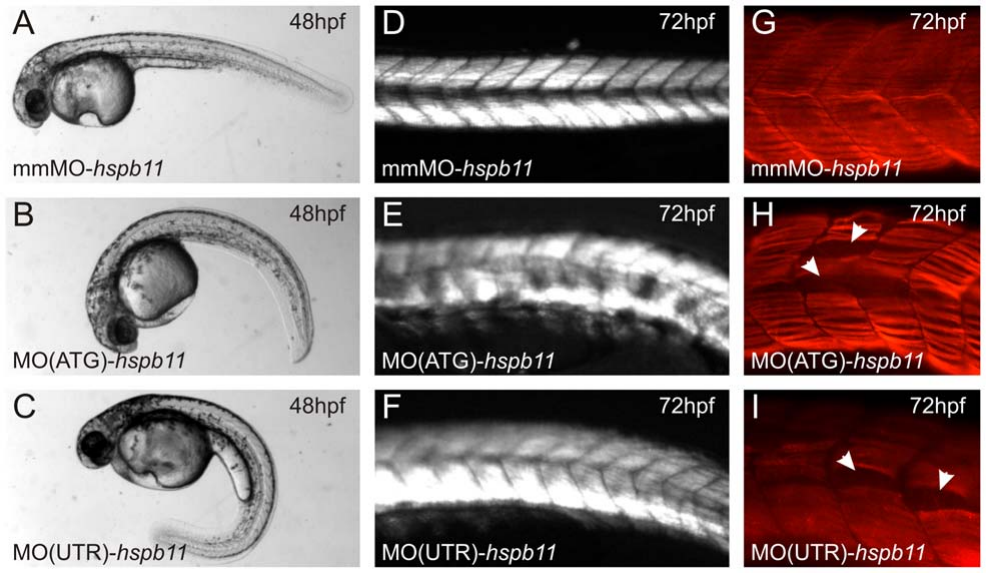
Knockdown of Hspb11 results in slow muscle myosin disorganization in skeletal muscles. (A, D and G) mmMO-*hspb11* injected embryos. (B, E and H) MO(ATG)-*hspb11* morphants. (C, F and I) MO(UTR)-*hspb11* morphants. (A–C) Phenotypic observations of morpholino injected embryos at 48 hpf. (D–E) Muscle organization determined with birefringence at 72 hpf. (G–I) Slow muscle myosin distribution at 72 hpf shown by immunofluorescence staining with antibody F59. Arrows indicate gaps in fiber distribution.

Both MO(ATG)-*hspb11* and MO(5′UTR)-*hspb11* morphants showed a ventral body curvature. In MO(ATG)-*hspb11* and MO(5′UTR)-*hspb11* injected embryos, 82% (n = 79, 2 replicates) and 73% (n = 114, 2 replicates) showed a morphant phenotype, respectively. The MO(5′UTR)-*hspb11* phenotype was much stronger and mmMO-*hspb11* control injected embryos showed no effects (Figure 6A–C). Additionally, we tried to rescue this morphant phenotype by coinjection of a *hspb11* mRNA that is not targeted by MO(ATG)-*hspb11* and MO(5′UTR)-*hspb11*. Coinjection of the *hspb11* mRNA with either MO(ATG)-*hspb11* or MO(5′UTR)-*hspb11* reduced the ventral body curvature phenotypes to 53% (n = 98, 2 replicates) and 51% (n = 137, 2 replicates), respectively.

In MO(ATG)-*hspb11*, MO(5′UTR)-*hspb11* and mmMO-*hspb11* injected embryos, we detected a wildtype *eng2a* expression pattern in muscle pioneers at 18 hpf (Figure S5D and E). Furthermore, knockdown of Hspb11 did not lead to a decrease in birefringence (Figure 6D–F). However, 80% (n = 10) of *hspb11* morphants showed a disruption in slow muscle myosin distribution. Gaps were formed between slow myofibers in knockdown embryos (Figure 6G–I). There are no other impairments, such as extra long intersomitic myofibers, detached myofibers or defective myosepta. This indicates that the development of muscle pioneers is not dependent on Hspb11. Moreover, the myofibril organization in superficial muscle cells is affected in the *hspb11* morphants, which suggests that Hspb11 is involved in slow muscle myofibril organization and/or maintenance.

## Discussion

By a comparative toxicogenomic approach we demonstrated that the AChE inhibitor APM induced specific responses on the gene expression levels in zebrafish embryos. Other non-AChE inhibiting compounds (DMB and DNP) did not affect the same cluster of genes. Gene set enrichment analyses (GSEA) revealed APM affected genes as enriched in a couple of gene sets involved in neuronal/muscle structure and differentiation. The enrichment of genes within these gene sets particularly after 48 h of exposure might indicate subtle morphological changes caused by APM. This is supported by the birefringence analysis which revealed structural alterations in muscular tissue. It is known that the lack of AChE activity in zebrafish results in an increase of muscular activity and can cause a progressive myopathy [5], [8]. We showed that genes of the APM-specific cluster (*hspb11*, *pdlim3b* and *socs3a*) were differently expressed in response to various AChE inhibitors. Pdlim3b, also known as Alp-like, is a structural protein and a member of the PDZ/LIM protein family which are characterized by the presence of both, a PDZ and a LIM domain [27]. Their functions are related to actin anchorage in muscles and non-muscle cells. PDZ/LIM proteins are required for muscle development and maintenance as has been shown for zebrafish *pdlim7* [28]. During zebrafish development *pdlim3b* (*alp-like*) is expressed in myotomes [27]. Hence, changes in *pdlim3b* expression in response to AChE inhibitors might be related to early disturbances of muscle structure and differentiation. The comparison of the responses to different AChE inhibitors and 4-nitrophenol indicated that *socs3a* was not induced specifically by AChE inhibitors. *Socs3a* belongs to the suppressor of cytokine signaling (Socs) protein family. It negatively regulates cytokine signaling in order to prevent excessive cytokine signaling that may disturb the normal homeostasis and cellular functions [29], [30]. Hspb11 is a member of the small heat shock proteins (sHSPs) family, which is a subclass of HSPs characterized by their low molecular weight and presence of the conserved α-crystalline domain. Many sHSPs act to prevent or reverse improper protein associations in an ATP independent manner and they are expressed in nearly all species [31], [32]. In our microarray experiments, *hspb11* was the only significantly induced member of sHSPs in zebrafish embryos exposed to APM. Commonly, the chaperone function, i.e. the support of correct protein folding, is considered as major physiological role of sHSPs and is regarded as the reason for their induction by a variety of stress factors [33]. It has been shown that HSPs are involved in several other processes, such as actin regulation, intermediate filaments assembly, apoptosis signaling or regulation of the cellular redox status [34], [35]. Furthermore, various diseases are known to be linked to mutations of sHSPs including cancer, neurodegenerations, and myopathies [36], [37]. Up to now, thirteen sHSPs are identified in zebrafish and their developmental and heat shock induced expression patterns have been described. The expression of *hspb11* is strongly induced after heat shock and transcripts are found throughout the somites and the hearts [38]. *Hspb11* orthologs seem to exist in nearly all vertebrates except mammals.

ACh is a neurotransmitter which is released by excitation of motoneurons at the neuromuscular junction. Binding of acetylcholine to nAChR depolarizes membranes of adjacent muscle cells and induces an intracellular calcium increase. Calcium acts as a messenger linking excitation events at the membrane with downstream effects, like contraction, ATP production and transcription [39], [40]. Thus, the observed altered expressions of *hspb11* could be mediated via the ACh/nAChR/calcium pathway. In this case, transcriptional up-regulation would primarily be observed in skeletal muscle tissues. An inactivation of nAChR would result in a loss of *hspb11* expression and *ache* mutants should exhibit elevated *hspb11* levels as well. Furthermore, it would be likely that compounds, which do not activate nAChR but increase cytosolic calcium levels, induce the expression of *hspb11*. Indeed, the experimental observations clearly support the anticipated role of nAChR and calcium, i.e. the *ache* −/− mutant exhibited an elevated *hspb11* expression in muscle tissue similar as in embryos exposed to APM. In *sop^fixe^−/−* mutants, which do not express a functional nAChR, the *hspb11* developmental expression pattern was completely lost and an induction of *hspb11* with APM was not possible. This data and the repressed transcription of *hspb11* by MS-222 provided evidences that developmental *hspb11* expression is muscle activity dependent.

Modulation of intracellular calcium levels with thapsigargin or caffeine, which most likely affect the whole zebrafish embryo and is not restricted to muscles, resulted in *hspb11* induction. Calcium dependent signal transduction cascades play important roles in the control of skeletal muscle gene expression in mammals and increased calcium levels result in, for instance, suppression of gene expression of myogenin and nAChR [41]. Likewise, in *ache* −/− mutant zebrafish embryos the sub-cellular localization of nAChRs is disturbed [5]. In our study, however, we did not detect down-regulation of muscle-specific genes such as nAChR or myogenin. It has been shown in zebrafish that acetylcholine and calcium signaling are required for the organization of slow muscle myofibrils [42]. Additionally, zebrafish mutants that exhibit disturbed calcium regulation in muscle tissues, such as *ache*, *sop^fixe^* or *acc*, showed disarrayed muscle fibers [5], [18], [43]. In our study, we showed that *hspb11* expression is modulated in *ache* and *sop^fixe^* mutants. A continuous elevation of calcium levels and subsequently enhanced calcium-activated protease activity has been shown to cause myofibre degeneration [20], [44]. This suggests that the correct regulation of cytosolic calcium transients is important for *hspb11* gene expression and myofibril organization and Hspb11 may compensate for calcium-induced protein degradation. We demonstrate that down-regulation of Hspb11 has no effect on muscle pioneer development, which is supported by the *eng2a* expression. Furthermore, morpholino knockdown of Hspb11 alters the distribution of slow muscle myosin. Hence, Hspb11 is important for myofibril organization and/or maintenance. Hspb11 may play a critical role in folding and assembly of various sarcomeric proteins during myofibrillogenesis and it remains to identify Hspb11 client proteins.

The transcriptional regulation of *hspb11* can be mediated via specific transcription factors, e.g. Creb, Nfat, Nf-κB, which are important components of intracellular signaling pathways and are regulated by calcium [45], [46]. By analyzing sequences 5 kb upstream of the *hspb11* transcriptional start site, several putative binding sites of these transcription factors are present (27 for Creb, 12 for Nfat, and 5 for Nf-κB, respectively, data not shown). However, the precise mechanism of the *hspb11* transcriptional regulation remains to be further investigated.

In summary, the combination of comparative toxicogenomics and functional analysis has led to the identification of *hspb11* as a possible marker for interference with neuromuscular signaling and/or calcium signaling. Further research need to address how elevated calcium levels and *hspb11* expression are linked.

## Materials and Methods

### Ethics Statement

All zebrafish husbandry and experimental procedures were performed in accordance with the German animal protection standards and were approved by the Government of Saxony, Landesdirektion Leipzig, Germany (Aktenzeichen 75-9185.64). Based on the *Guidelines on the protection of experimental animals* by the Council of Europe, Directive 2010/63/EU, which allows zebrafish embryos to be used up to the moment of independent feeding (approximately 5 days after fertilization). Because embryos used here were no more than 4 days old, no license is required by Council of Europe (1986), Directive 2010/63/EU or the local authority.

### Fish maintenance

A wildtype zebrafish strain (WiK) was obtained from the Max Planck Institute of Developmental Biology (Tübingen, Germany) and cultured at 26°C according to standard procedures [47]. Mutant lines *ache* ^sb55^, *sop^fixe^* and *smu* ^b577^ have been described previously and were kept at the Institute of Toxicology and Genetics, Karlsruhe [5], [18], [48].

### Chemical exposure experiments

Embryos were exposed from 2–50 hpf, 26–50 hpf, 48–50 hpf and/or 12 hpf (5-somite stage) to 72 hpf in 100 ml crystallization dishes covered with watchmaker glasses with the chemicals. The following chemicals were used: APM (azinphos-methyl, CAS#86-50-0, purity 98,5%, Fluka), DMB (1,4-dimethoxybenzene, CAS#150-78-7, purity 99%, Sigma-Aldrich), 2,4-DNP (2,4-dinitrophenol, CAS#51-28-5, purity 99%, Fluka), GAL (galantamine hydrobromide, CAS#1953-04-4, purity 98%, Sigma), propoxur (CAS#114-26-1, purity 99,9%, Fluka), disulfoton (CAS#298-04-4, purity 99%, Fluka), 4-nitrophenol (CAS#100-02-7, Fluka), thapsigargin (CAS#67526-95-8, purity ≥98%, Sigma), caffeine (CAS#58-08-2, purity ≥99%, Fluka) and MS-222 (Ethyl 3-aminobenzoate methanesulfonate, CAS#886-86-2, purity 98%, Sigma-Aldrich). Stock solutions of the chemicals were prepared either in exposure medium (294.0 mg/L CaCl_2_*2H_2_O, 123.3 mg/L MgSO_4_*7H_2_O, 64.7 mg/L NaHCO_3_, 5.7 mg/L KCl; according to ISO guideline 15088 or DMSO (thapsigargin)). Exposure concentrations were prepared by dilution of stock solutions. In case of thapsigargin DMSO concentration in exposure and control media was adjusted to 0.01%. Exposure concentrations of APM, DMB and DNP were analyzed by GC-MS by the TZW (Technologie Zentrum Wasser, Karlsruhe, Germany). After 48 h of exposure 90% (APM, 5.23 µmol/L), 75% (DMB, 379 µmol/L) or 74% (DNP, 10.6 µmol/L), respectively, of the nominal concentrations could be detected.

### Microarray analysis

RNA was extracted from 50 embryos for each treatment (controls and embryos exposed to the LC_10_, 4 independent biological replicates each). We used the 4×44 K Agilent *D. rerio* oligo microarrays (Amadid #015064, Agilent, Böblingen, Germany). The synthesis/labeling of cRNA with Cy3 (Agilent Low RNA Input Linear Amplification Kit) and hybridizations with the microarray slides were performed according to the manufacturer's instructions. Slides were scanned with an Agilent DNA Microarray Scanner.

Fluorescent intensities of individual microarray spots were extracted using the Agilent Feature Extraction software (Version 9.1). Log2-transformed fluorescent intensities were quantile normalized and used to calculate the ratio for each treatment versus the mean fluorescent value of the respective control. Analysis of statistical significance with SAM [49], false discovery rate adjusted to 0, p<0.03) and hierarchical clustering were performed with these ratios and the TMEV software package version 4.3 [www.tm4.org] [50]. The microarray data is MIAME compliant, and raw and normalized data have been submitted to the Gene Expression Omnibus (GEO) database [www.ncbi.nlm.nih.gov/geo/query/acc.cgi?acc=GSE27680].

For the identification of biological functions and pathways associated with the changes in gene expression caused by the APM treatment, Gene Set Enrichment Analyses (GSEA) [www.broadinstitute.org/gsea/index.jsp] [51], [52] was performed. Since the Molecular Signatures Database (MSigDB), on which GSEA is based, contains mostly human and rodent gene sets, we used the human ortholog gene names/annotations for this analysis. Human orthologs of the whole microarray probe set were assigned using Biomart [http://www.ensembl.org/biomart/index.html]. GSEA was performed using the complete dataset whereas 5961 genes, i.e. those for which a human ortholog was identified, could be associated with gene sets from the database. The gene sets of the MSigDB (v3.0) databases C2 (Gene sets collected from various sources such as online pathway databases, publications in PubMed, and knowledge of domain experts) and C5 (Gene sets of Gene Ontology terms) were used for the analysis.

### AChE activity assay

Zebrafish embryos were exposed with different APM concentrations for 24 h (26–50 hpf). 30 embryos were used for AChE activity assay and were performed as previously described [53]. Non-linear modeling was performed from the plot of AChE activities with JMP 8.0 software (SAS, Cary, NC).

### Quantitative real-time PCR analysis

Total RNA was extracted from 50 control, mutant or exposed zebrafish embryos at 50 hpf. 2 µg of total RNA was reversely transcribed with RevertAid™ H Minus Reverse Transcriptase (Fermentas) according to the manufacturer instructions. qPCR was carried out using Step-One-Plus PCR System (Applied Biosystems) and we used the SensiMix^™^ SYBR with ROX as passive reference (Bioline). qPCR experiments were performed with three independent biological replicates and each single run was performed in three replicate samples. Statistical analysis of qPCR experiments were performed with one-way ANOVA followed by Dunnett's post test using GraphPad Prism 5.0 software (GraphPad Software, San Diego California USA). Primer sequences are given in Table S1. Relative expression levels were determined by using the ΔΔCt method [54].

### Cloning, *in situ* hybridization, immunohistochemistry, and microscopy

The open reading frame (ORF) of *hspb11* was amplified with ORF primers (Table S2), cloned into pCRII-blunt vector (Invitrogen) and was verified by sequencing. This construct was used for the synthesis of *in situ* hybridization probes. GFP constructs were amplified with F1-MOATG-gfp/R1-GFP primers for *atg-gfp* and F2-MOUTR-gfp/R1-GFP primers for *utr-gfp* (Table S2) using pEGFP-N1 (Clonetech) vector as template. Construct for the rescue experiment was generated by using mm*hspb11*-F/*hspb11*-ORF_Rev primers (Table S2) and *hspb11*-ORF-pCRII vector as template. Products were cloned into pCRII blunt vector (Invitrogen) and subcloned into pCS2P+. We performed whole-mount *in situ* hybridization (WISH) and immunohistochemistry as described previously [55], [56]. We used the monoclonal antibody directed against slow muscle myosin (F59). The F59 monoclonal antibody developed by Frank E. Stockdale was obtained from the Developmental Studies Hybridoma Bank developed under the auspices of the NICHD and maintained by The University of Iowa, Department of Biology, Iowa City, IA 52242. WISH was performed with ∼50 embryos per treatment if not stated elsewhere. Birefringence was analyzed with a stereomicroscope (MZ16F; Leica, Wetzlar, Germany) as described previously [5]. A Leica compound microscope (DM 5000B) and LCS software (Leica, Wetzlar, Germany) were used to analyze antibody staining.

### Morpholino knockdown and mRNA injection

Morpholinos (Gene Tools, LLC, Philomath, OR) were dissolved in water and injected at the following concentrations: 0,25 mM – 1 mM MO(ATG)-*hspb11* (5′- TCGGGCAAAGCATCTTCAGTGGATT- 3′); 0,25 mM–1 mM mmMO-*hspb11* (5′-TCcGGgAAAGgATCTTCAcTGcATT-3′) was a 5 base mismatch control for MO(ATG)-*hspb11*; 0,25 mM–0,5 mM MO(5′UTR)-*hspb11* (5′- TTTGCTGTTGAGCTGTTTGGCTTCT- 3′); 0,25 mM–1 mM MO-*p53* (5′-GACCTCCTCTCCACTAAACTACGAT-3′). 5′ capped mRNAs were transcribed from linearized vectors using the mMESSAGE mMACHINE SP6 Kit (Applied Biosystems/Ambion, Austin, TX). Injection needles were pulled from borosilicate glass capillary tubes with filament (GC100F-10; Warner Instruments, LLC, Hamden, CT) using a Narishige micropipette puller (Tokyo, Japan). Embryos were injected with the Eppendorf FemtoJet (Hamburg, Germany) through the chorion into the yolk compartment at the one-cell stage.

## Supporting Information

Figure S1 **Acute toxicity of AMP, DMB and DNP in zebrafish embryos.** Dose response curves were determined by recording mortality in 2–50 hpf exposed embryos. Non linear regression modeling was performed with SigmaPlot version 11 (Systat Sofware Inc., San Jose, California) using the Hill 4 parameter equation (f = y0+a*x∧b/(c∧b+x∧b)).
(TIF)

Figure S2 **AChE enzymatic activity in response to various APM concentrations.** AChE enzyme activities are expressed as fold change of controls (based on specific activity). (n  =  3, exposures from 26–50 hpf).
(TIF)

Figure S3 **Heat map of a hierarchical cluster analysis of genes significantly differentially expressed in zebrafish embryos.** Embryos were exposed for 24 h (26–50 hpf) or 48 h (2–50 hpf) to azinphos-methyl (APM), 1,4-dimethoxybenzene (DMB) or 1,2-dinitrophenol (DNP). Genes with significantly altered expression were identified using SAM (TM4 software suite). Treatments were performed by two separate series of experiments, one for the 24 h exposure and one for the 48 h exposure. C24 and C48 are control samples that refer to these separate experiments. Data represent the log2-ratio of each treatment or control to the average of control levels (either C24 or C48). Numbers 1–4 indicate different independent biological replicates. The cluster of genes, which are specifically regulated by APM, is marked by orange lines.
(TIF)

Figure S4 **qPCR analysis of** ***hspb11*** **,** ***pdlim3b*** **and** ***socs3a*** **expression in zebrafish embryos.** Embryos were exposed from 26–50 hpf to different AChE inhibitors. (A) Propoxur, (B) disulfoton, and (C) galantamine. (D) 4-nitrophenol served as unspecific (non-acetylcholinesterase inhibiting) control. Concentrations are given in µM if not differently labeled. Bars represent the relative gene expression as fold change of the respective untreated control as mean ± standard deviation of three replicate exposures. Control  =  ctrl. * P<0.05.
(TIF)

Figure S5 **Muscle pioneer specific expression analysis of** ***hspb11*** **and** ***eng2a*** **expression**. (A) Transcripts of *hspb11* mRNA are localized in muscle pioneers of wildtype or *smu* heterozygous embryos (+/?). (B) *smu*−/− embryos lack muscle pioneers and *hspb11* expression is absent. (C) Homozygous *sop^fixe^−/−* mutants show wildtype *eng2a* expression in muscle pioneers and at the midbrain-hindbrain boundary (mhb). (D and E) Muscle pioneer development in MO(UTR)-*hspb11* and mmMO-*hspb11* injected embryos was not effected, confirmed by *eng2a* expression.
(TIF)

Figure S6 **MO(ATG)-** ***hspb11*** **and MO(5′UTR)-** ***hspb11*** **morpholinos specifically target their** ***hspb11*** **binding sites.** Embryos injected with *gfp* mRNA that contains the MO binding site expresses GFP. Co-injection of MO(ATG)-*hspb11* or MO(5′UTR)-*hspb11* with the *gfp* mRNA abolishes GFP expression, whereas with the mmMO-*hspb11* morpholino does not reduce GFP expression. *atg-gfp* mRNA contains MO(ATG)-*hspb11* binding site. *utr-gfp* mRNA contains MO(UTR)-*hspb11* binding site.
(TIF)

Table S1 **Gene sets exhibiting greatest overlap to differentially expressed genes in APM treated zebrafish embryos.** The analysis was performed by the software GSEA (gene set enrichment analysis) for all genes of which a human ortholog could be identified. Gene expression patterns of APM-treatments were compared to all other treatments (controls, exposure to 1,4-dimethoxybenzene and 1,2-dinitrophenol). Only gene sets with a p-value<0.01 are shown.
(XLS)

Table S2 **List of primers used in this study.**
(XLS)
