# Supplementary figures and images for: Transcriptional Response of Zebrafish Embryos Exposed to Neurotoxic Compounds Reveals a Muscle Activity Dependent hspb11 Expression
Source: PLoS One. 2011 Dec 19;6(12):e29063. doi: 10.1371/journal.pone.0029063 (PMC3242778; doi:10.1371/journal.pone.0029063)

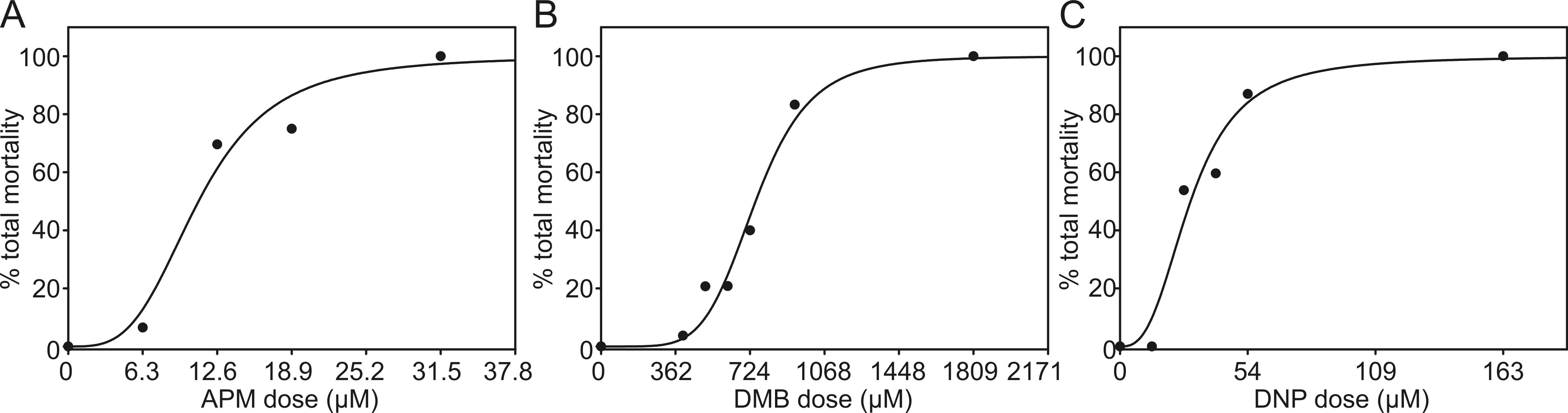

Supplement: Figure S1 — Acute toxicity of AMP, DMB and DNP in zebrafish embryos. Dose response curves were determined by recording mortality in 2–50 hpf exposed embryos. Non linear regression modeling was performed with SigmaPlot version 11 (Systat Sofware Inc., San Jose, California) using the Hill 4 parameter equation (f = y0+a*x∧b/(c∧b+x∧b)). (TIF) [file pone.0029063.s001.tif]

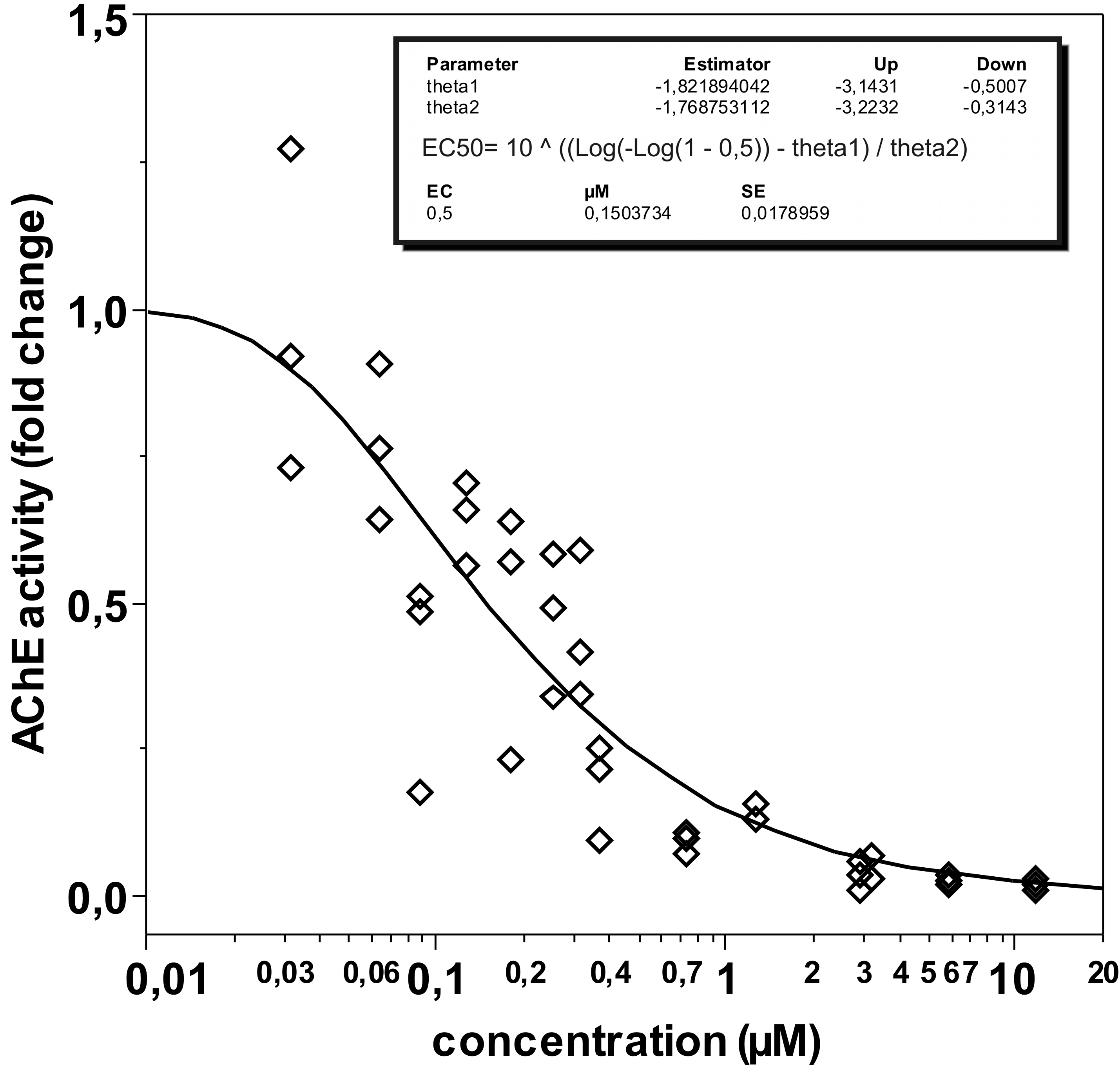

Supplement: Figure S2 — AChE enzymatic activity in response to various APM concentrations. AChE enzyme activities are expressed as fold change of controls (based on specific activity). (n = 3, exposures from 26–50 hpf). (TIF) [file pone.0029063.s002.tif]

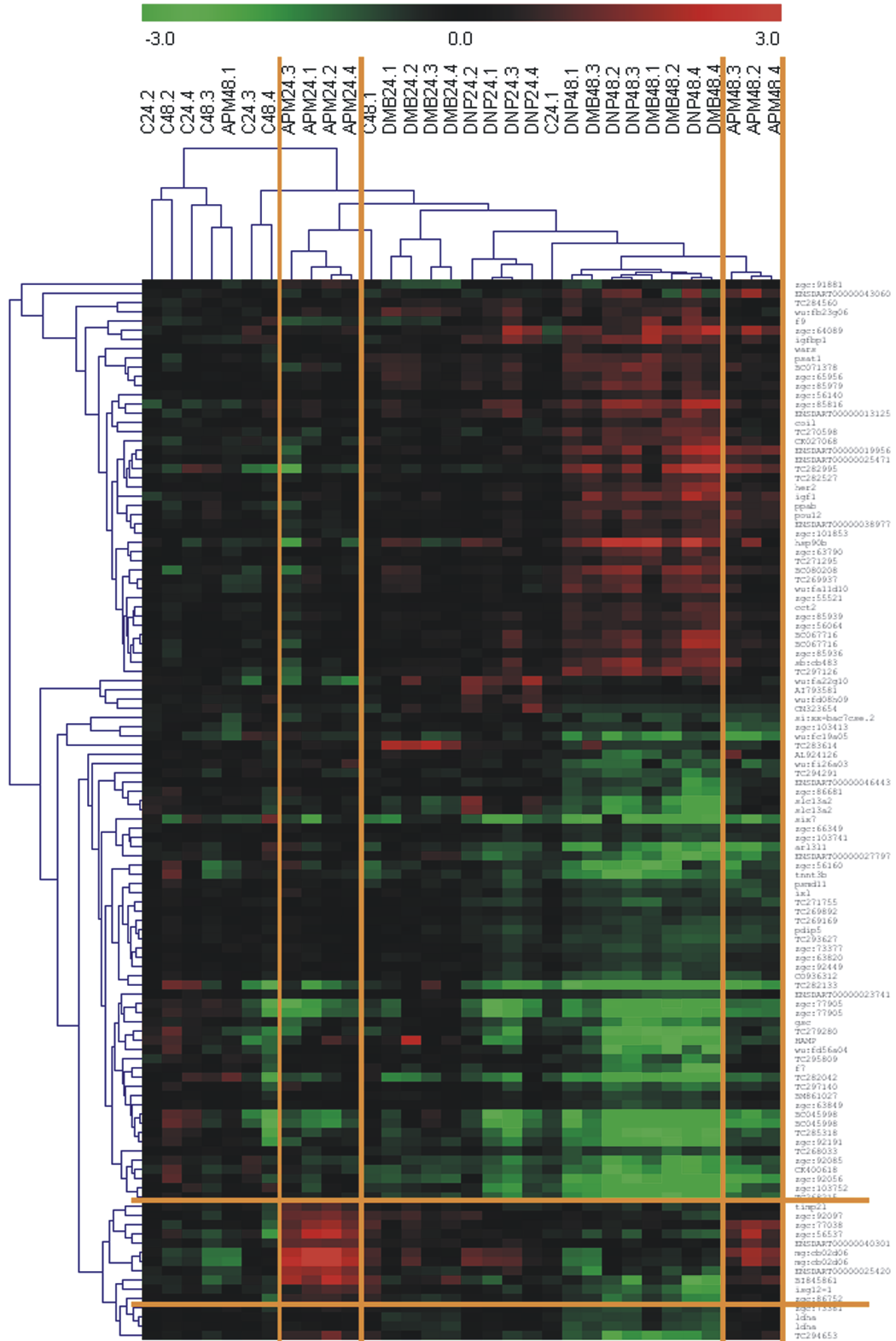

Supplement: Figure S3 — Heat map of a hierarchical cluster analysis of genes significantly differentially expressed in zebrafish embryos. Embryos were exposed for 24 h (26–50 hpf) or 48 h (2–50 hpf) to azinphos-methyl (APM), 1,4-dimethoxybenzene (DMB) or 1,2-dinitrophenol (DNP). Genes with significantly altered expression were identified using SAM (TM4 software suite). Treatments were performed by two separate series of experiments, one for the 24 h exposure and one for the 48 h exposure. C24 and C48 are control samples that refer to these separate experiments. Data represent the log2-ratio of each treatment or control to the average of control levels (either C24 or C48). Numbers 1–4 indicate different independent biological replicates. The cluster of genes, which are specifically regulated by APM, is marked by orange lines. (TIF) [file pone.0029063.s003.tif]

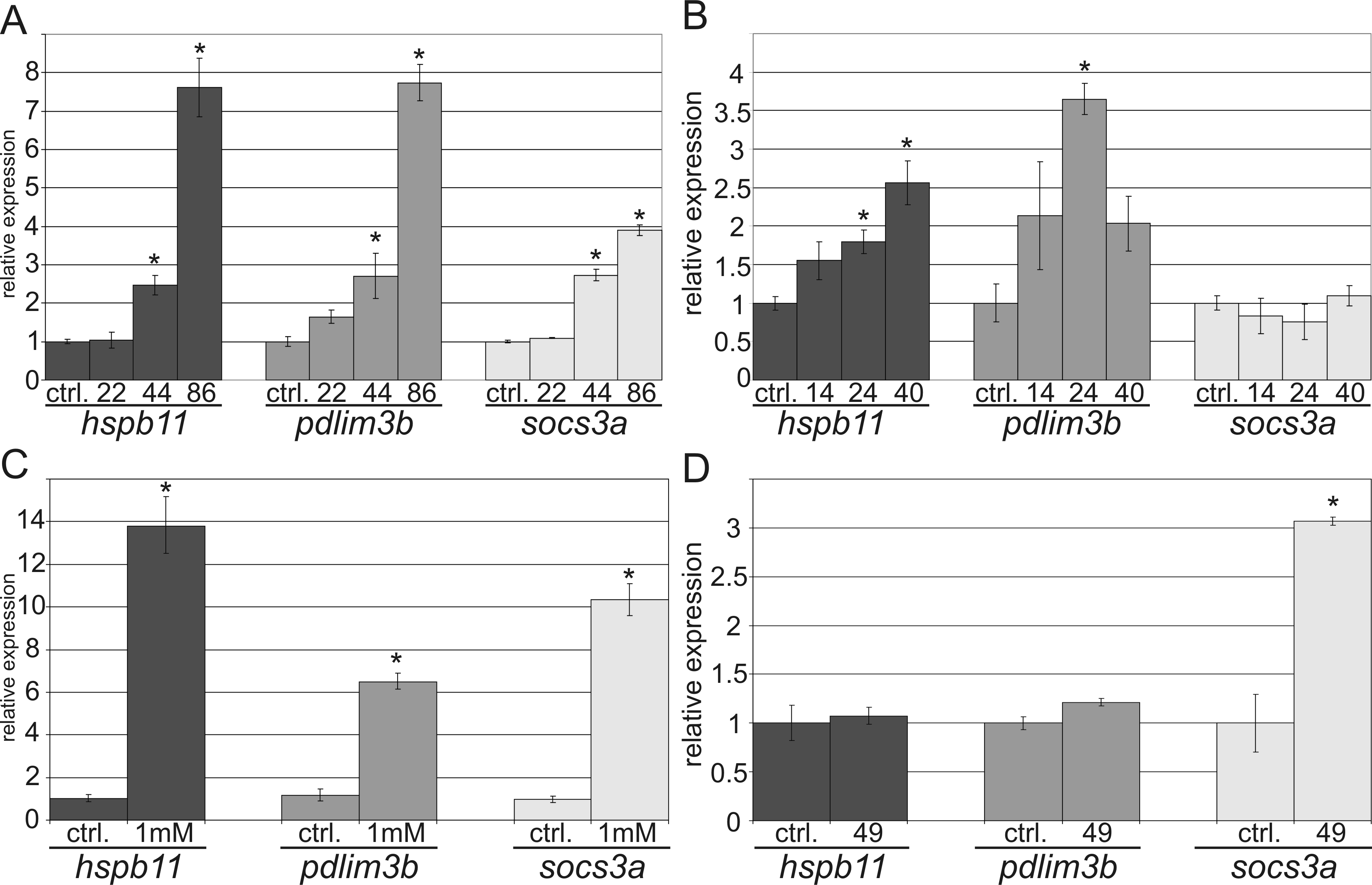

Supplement: Figure S4 — qPCR analysis of hspb11 , pdlim3b and socs3a expression in zebrafish embryos. Embryos were exposed from 26–50 hpf to different AChE inhibitors. (A) Propoxur, (B) disulfoton, and (C) galantamine. (D) 4-nitrophenol served as unspecific (non-acetylcholinesterase inhibiting) control. Concentrations are given in µM if not differently labeled. Bars represent the relative gene expression as fold change of the respective untreated control as mean ± standard deviation of three replicate exposures. Control = ctrl. * P<0.05. (TIF) [file pone.0029063.s004.tif]

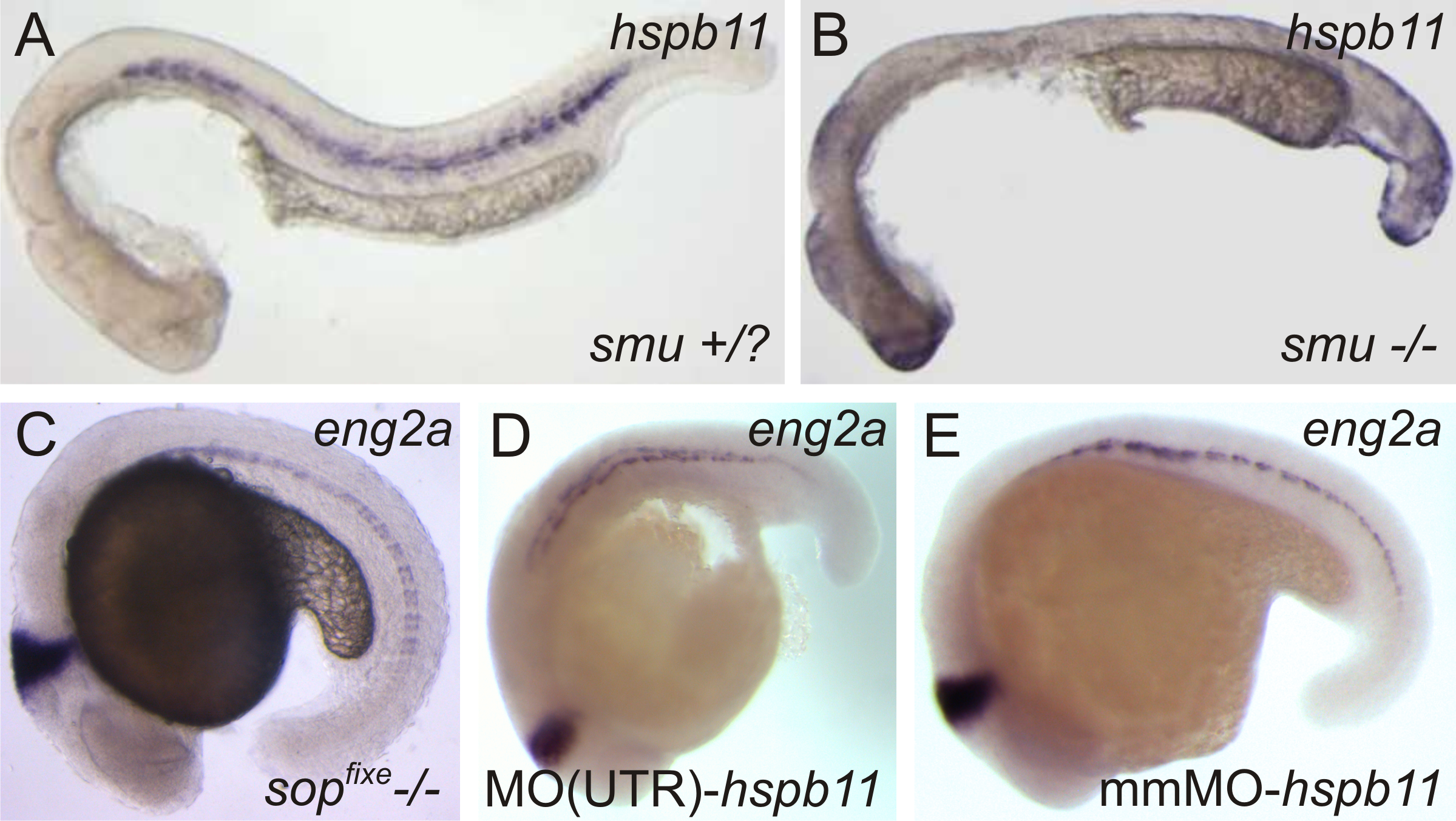

Supplement: Figure S5 — Muscle pioneer specific expression analysis of hspb11 and eng2a expression. (A) Transcripts of hspb11 mRNA are localized in muscle pioneers of wildtype or smu heterozygous embryos (+/?). (B) smu−/− embryos lack muscle pioneers and hspb11 expression is absent. (C) Homozygous sopfixe−/− mutants show wildtype eng2a expression in muscle pioneers and at the midbrain-hindbrain boundary (mhb). (D and E) Muscle pioneer development in MO(UTR)-hspb11 and mmMO-hspb11 injected embryos was not effected, confirmed by eng2a expression. (TIF) [file pone.0029063.s005.tif]

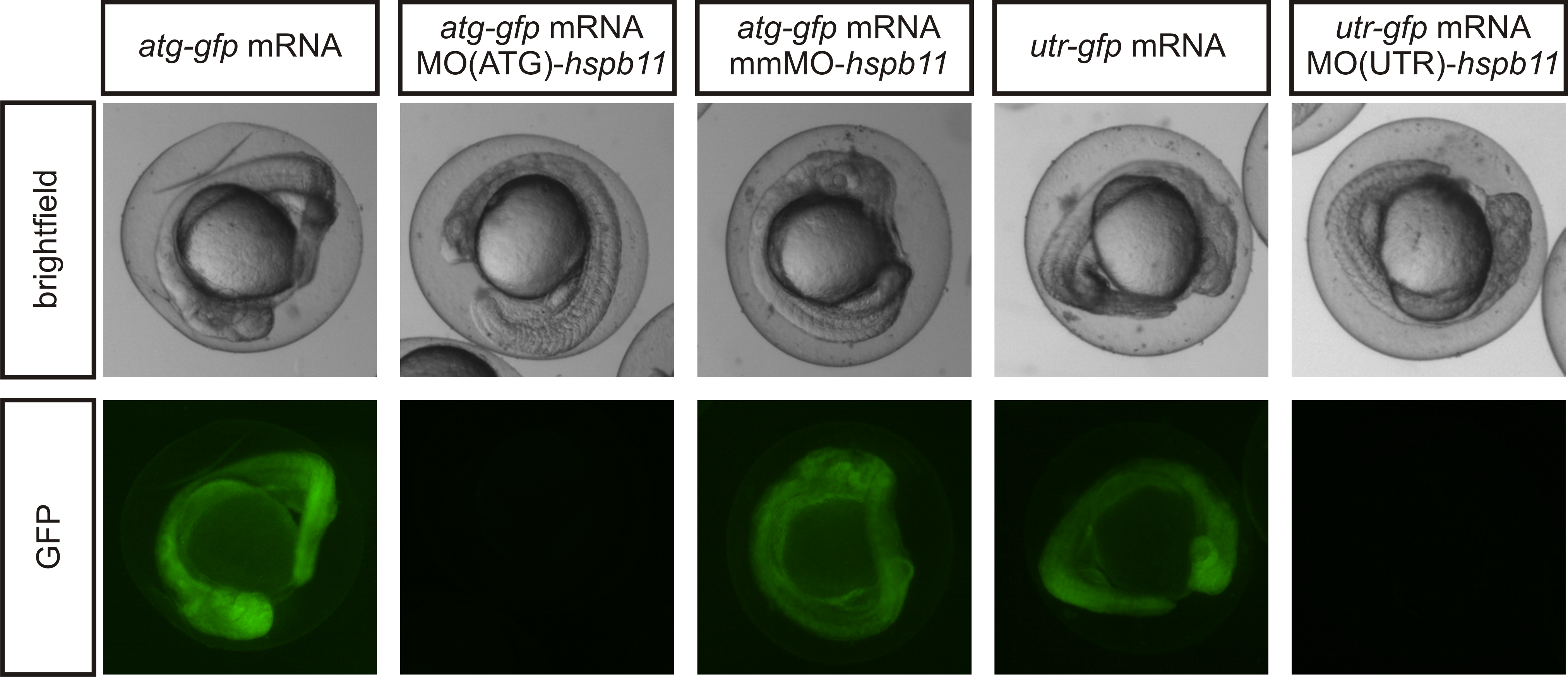

Supplement: Figure S6 — MO(ATG)- hspb11 and MO(5′UTR)- hspb11 morpholinos specifically target their hspb11 binding sites. Embryos injected with gfp mRNA that contains the MO binding site expresses GFP. Co-injection of MO(ATG)-hspb11 or MO(5′UTR)-hspb11 with the gfp mRNA abolishes GFP expression, whereas with the mmMO-hspb11 morpholino does not reduce GFP expression. atg-gfp mRNA contains MO(ATG)-hspb11 binding site. utr-gfp mRNA contains MO(UTR)-hspb11 binding site. (TIF) [file pone.0029063.s006.tif]
